# Recognition-Element-Driven Rapid Detection of Biogenic Amines in Foods: From Molecular Recognition to On-Site Sensing

**DOI:** 10.3390/foods15152741

**Published:** 2026-08-04

**Authors:** Jing Wang, Ruoxi Zhang, Mengyao Chen, Yixuan Wang, Huilin Liu, Huijuan Yang

**Affiliations:** 1 Key Laboratory of Digital-Intelligence and Dynamic Perception for Food Quality of China Light Industry, Beijing Technology and Business University, Beijing 100048, China; 2 Beijing Laboratory for System Engineering of Carbon Neutrality, Beijing Municipal Education Commission, Beijing 100048, China

**Keywords:** biogenic amine, recognition element, on-site testing, biosensors, food safety

## Abstract

Biogenic amines (BAs) are nitrogenous compounds formed by microbial decarboxylation of amino acids in protein-rich foods. Their accumulation indicates spoilage and poses health risks. Traditional methods like high-performance liquid chromatography (HPLC) and gas chromatography (GC) are sensitive but time-consuming, limiting on-site use. Rapid technologies based on specific recognition molecules offer feasible alternatives for real-time monitoring. This review summarizes five categories of recognition elements: antibodies, aptamers, molecularly imprinted polymers (MIPs), enzymes, and peptides for BA detection in foods. These elements convert BA concentrations into optical, electrical, or colorimetric signals, establishing a complete biosensing chain. Integration with portable platforms (lateral flow assays (LFAs), microfluidic chips, smart labels, and smartphone devices) is also discussed. Recognition-element-based sensing enables high-selectivity and rapid monitoring of BAs in foods. Antibody/aptamer systems excel in specific histamine detection, enzyme platforms in rapid total amine assessment, and MIPs in chemical stability and matrix tolerance. Yet practical application is limited by poor selectivity for similar amines, matrix interference, insufficient real-food validation, and device standardization. Our future focus will be on AI-assisted design, multi-target arrays, smartphone quantification, and IoT-enabled freshness monitoring.

## 1. Introduction

Biogenic amines (BAs) are a group of nitrogenous organic compounds commonly detected in high-protein foods like seafood, meat, and fermented products. Major BA species include histamine, tyramine, putrescine, cadaverine, and spermidine, which are synthesized through microbially induced amino acid decarboxylation [1]. Seafood rich in histidine is prone to histamine accumulation under acidic anaerobic conditions, whereas tyramine predominantly occurs in fermented vegetables and dairy products due to the metabolic activity of lactic acid bacteria. In contrast, meat spoilage is mainly characterized by the buildup of putrescine and cadaverine, which serve as early indicators of protein degradation [2]. Because of its toxic effects, histamine is strictly regulated for food safety purposes. The U.S. Food and Drug Administration (FDA) sets a limit of 50 ppm for histamine in fish products, while countries such as South Korea and China recommend limits of 200–400 mg/kg for histamine in fermented foods, and tyramine levels are often used as spoilage indicators for fermented products [3]. According to Chinese national standards (GB), mandatory maximum limits for other BAs (e.g., putrescine, cadaverine, tryptamine, and spermidine) are generally not established, except that tyramine is restricted to ≤100 mg/kg in fermented meat products. A comparison of the common BAs, their regulatory limits, the main food matrices, and the typical detection technologies is presented in Table 1.

Primarily generated via the decarboxylation of free amino acids through the enzymatic action of spoilage or fermentative microorganisms, BAs are key indicators for evaluating the freshness and safety of aquatic products [4]. Certain BAs occur naturally at low levels, but the accumulation of histamine, tyramine, putrescine, and cadaverine in protein-rich foods indicates microbial growth and severe spoilage. Volatile amines cause off-flavors, while non-volatile ones pose health risks upon excessive intake: histamine can trigger allergic-like poisoning (e.g., headache and hypotension), tyramine is associated with hypertensive crises, and putrescine and cadaverine, though less toxic alone, enhance overall toxicity by inhibiting histamine-metabolizing enzymes. Thus, monitoring BA concentrations provides reliable indicators for food freshness, quality, and safety.

Common chromatographic methods include thin-layer chromatography (TLC), high-performance liquid chromatography (HPLC), gas chromatography (GC), ion chromatography (IC), and capillary electrophoresis (CE) [5]. Researchers rely on various traditional separation approaches to quantify BAs. While HPLC delivers favorable selectivity and sensitivity, it demands tedious derivatization and considerable expenditure. GC completes analysis quickly, at the cost of weakened accuracy and reliability. TLC is affordable and easy to operate for parallel testing, yet it struggles with unsatisfactory quantification performance and low reproducibility. IC removes the need for derivatization and eases extraction, though its sample pretreatment remains intricate. Due to the similar molecular masses of target substances, CE also suffers from insufficient sensitivity despite simplified operational workflows [6].

Extended global food logistics increases food spoilage risks. Standard laboratory assays are accurate yet slow, expensive, and device-reliant, making them impractical for field screening. Hence, low-cost and user-friendly rapid detection tools are highly required for food safety management. Recently, nanomaterials have been combined with recognition components to enhance sensor performance. Furthermore, intelligent hardware and artificial intelligence have optimized detection systems, realizing intelligent identification and chain-wide monitoring to achieve full food safety regulation.

Different from existing reviews that mainly categorize BA biosensors according to detection techniques such as optical or electrochemical methods, this article offers a systematic and comparative analysis centered on recognition elements. It evaluates five types of recognition materials including antibodies, enzymes, molecularly imprinted polymers (MIPs), nucleic acid aptamers, and peptides. The discussion focuses on their distinct interaction mechanisms, signal conversion modes, and integration into emerging detection platforms such as smartphone-assisted systems, microfluidic chips, and paper-based substrates. This recognition-oriented perspective highlights the importance of selecting and designing highly specific and stable recognition units for real-time applications. The presented insights contribute to advancing food quality assessment, freshness monitoring, food safety assurance, and supply chain surveillance. Furthermore, this review provides practical references for selecting suitable recognition elements and detection platforms tailored to different food scenarios, including fresh meat, aquatic products, dairy goods, and fermented foods, thereby facilitating scenario appropriate deployment in routine quality control and field inspection.

## 2. Recognition Elements for Rapid Detection of BAs

Recognition elements serve as essential components for sensors and analytical systems. Their core function is to selectively bind target analytes and trigger specific reactions, converting molecular interactions into measurable signals. With the continuous progress of sensor technology and material engineering, recognition elements with high efficiency, stability, and selectivity have become an active research area [7].

### 2.1. Antibody-Based Recognition Systems

#### 2.1.1. Application of Traditional Monoclonal/Polyclonal Antibodies in Immunoassay

**Figure 1:**
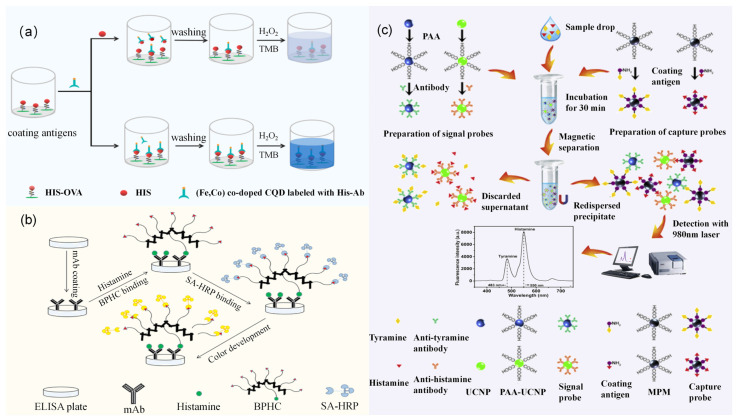
(**a**) An ic-ELISA relying on an Fe–Co co-doped carbon dot-tagged anti-histamine antibody as a nanozyme for histamine monitoring in fish tissues. (Reprinted with permission from ref. [10]. Copyright 2021, Elsevier.) (**b**) A high-sensitivity sandwich ELISA using histamine-targeted mAbs to screen histamine within various seafood including fish, shrimp, and crab. (Reprinted with permission from ref. [14]. Copyright 2021, Elsevier.) (**c**) A dual-antibody-based fluorescent immunoassay utilizing upconversion nanoparticles for the simultaneous quantification of tyramine and histamine in diverse food matrices. (Reprinted with permission from ref. [15]. Copyright 2020, Elsevier.).

Monoclonal antibodies (mAbs) were first developed based on hybridoma technology proposed by Köhler and Milstein in 1975 [8]. This approach fuses immunized B lymphocytes with myeloma cells, followed by screening to filter cell strains capable of stable mAb secretion. Such homogeneous antibodies can specifically bind to only one epitope of the target antigen [9]. In the context of BA detection, mAbs have been developed against key targets such as histamine, tyramine, and putrescine. The mAbs can provide high target specificity when well-designed haptens and screening strategies are available; however, the discrimination of structurally simple aliphatic amines remains challenging. Li et al. [10] constructed an indirect competitive enzyme-linked immunosorbent assay (ELISA) that utilized iron–cobalt co-doped carbon dots as nanozyme labels for histamine antibodies (Figure 1a). This method can sensitively and specifically identify histamine in fish matrices, with its limit of detection (LOD) reaching 0.50 mg/kg.

Polyclonal antibodies (pAbs) are extracted and purified from the serum of antigen-immunized animals. Derived from diverse B-cell lineages, these antibody mixtures are capable of binding to multiple epitopes on target antigens. In comparison with mAbs, pAbs require less preparation time and lower production cost while exhibiting superior binding affinity, thereby gaining extensive application in immunoassay platforms. In the context of BA analysis, pAbs have been successfully applied in enzyme-linked immunosorbent assays and immunosensors for rapid screening of histamine, tyramine, and putrescine in meat, fish, and fermented products. Their ability to recognize structural analogs with moderate cross-reactivity further facilitates simultaneous monitoring of multiple BAs, which is particularly valuable for food freshness evaluation and supply chain quality control. Sheng W et al. [11] established an indirect competitive ELISA (icELISA) relying on pAbs for tyramine quantification in meat and seafood. This method obtained an LOD of 0.02 mg/L in standard solutions and 1.20 mg/kg in actual food samples, with negligible cross-reactivity against tyrosine and other BAs. Even so, conventional pAbs still have inherent drawbacks in complicated food matrices. Matrix disturbances, poor denaturation resistance, and shielded epitopes may weaken their practical sensitivity and specificity during food detection.

#### 2.1.2. Advantages of Nanobodies in Complex Matrix Detection

Nanobodies are single-domain antibodies composed of heavy-chain variable regions. They show strong potential for detecting targets in complex matrix samples [12]. Owing to their excellent physicochemical stability and modifiable properties, nanobodies tolerate high temperatures, extreme pH values, and organic solvents. These characteristics simplify sample pretreatment and facilitate the construction of reusable sensing interfaces. The single-gene structure also makes it easy to attach signal molecules through gene fusion or chemical conjugation. It also enables the preparation of multivalent or multispecific complexes to improve binding affinity and detection sensitivity.

These merits make nanobodies compatible with flow cytometry immunoassays, biosensors, and microfluidic chips, rendering them uniquely applicable for high-interference scenarios in food safety supervision and environmental monitoring. In practical application scenarios, such as the rapid on-site testing of seafood, meat, and fermented products, nanobodies maintain remarkable structural stability against the matrix variations induced by protein degradation and lipid oxidation. This robustness allows them to accurately capture small BA targets like histamine or tyramine within complex matrices, potentially reducing matrix-induced false responses compared with conventional antibodies. Consequently, nanobody-integrated devices are ideally suited for real-time freshness tracking across the food supply chain and high-throughput screening in resource-limited field settings.

#### 2.1.3. Antibody-Based Recognition Mechanism

As typical biological recognition molecules, antibodies are extensively adopted in affinity-dependent detection systems owing to their excellent affinity, specificity, and sensitivity toward target analytes [13]. Their core working principle relies on the selective noncovalent interaction between antibody antigen-binding fragments (Fab) and target molecules (amino or amine carrier protein conjugates), and the binding signals can be further converted into detectable readouts through signal transduction. To boost the detection sensitivity of small-molecule amines, common signal amplification strategies include binding amino groups to macromolecular carrier proteins and introducing enzyme labels to trigger catalytic reactions.

At the molecular interference level, lipids in the food matrix can non-specifically bind to the hydrophobic pocket regions of antibodies through hydrophobic interactions, distorting the three-dimensional conformation of the antigen-binding site; proteins can partially occupy the complementarity-determining regions via electrostatic adsorption or hydrogen-bond competition, hindering proper access of the target amines; and polyphenols and pigments can engage in non-specific recognition with aromatic residues within the binding pocket through π–π stacking and hydrogen-bond competition, increasing the risk of cross-reactivity. The cumulative effect of these interference mechanisms generally necessitates elaborate sample pretreatment to reduce matrix interference and ensure that detection sensitivity is not significantly compromised.

Based on the above detection mechanisms, Yang et al. [14] fabricated a competitive sandwich ELISA by combining polylysine-long-armed biotin–histamine complexes with a biotin–streptavidin amplification system (Figure 1b). This derivatization-free method enables the highly sensitive quantification of histamine in seafood, with a linear range of 11.7–1500 ng/mL and an LOD of 5.86 ng/mL. Moreover, Zhang et al. [15] designed a competitive fluorescence immunoassay for the synchronous detection of tyramine and histamine in food. This technique adopted two upconversion nanoparticles with distinct emission wavelengths as multicolor labels and integrated magnetic separation technology (Figure 1c). It obtained LODs of 0.1 μg/L for tyramine and 0.01 μg/L for histamine, showing much higher sensitivity than conventional ELISA protocols. The detection limits obtained by the above immunoassays (histamine: 5.86 ng/mL; tyramine: 0.1 μg/L; histamine: 0.01 μg/L) are at least four orders of magnitude lower than the regulatory maximum limits for histamine (50–200 mg/kg). Although tyramine lacks a unified mandatory limit, these values are also far below the generally recommended threshold (100–200 mg/kg). Thus, the sensitivity of these sensors not only reliably identifies non-compliant samples but also supports early-warning screening at trace levels, fully meeting the practical requirements for rapid food safety testing. Table 2 presents the sensing strategies based on antibody recognition systems for BA detection in foods.

#### 2.1.4. Antibody-Based Recognition Element Advantages, Limitations, and Application Scopes

Antibody-based sensing systems take advantage of high affinity and specificity to selectively bind BA haptens and reduce cross-reactivity, while signal amplification techniques support their trace-level detection. The mAb techniques enable standardized and large-scale production, thereby commercializing mature detection platforms such as ELISA and immunochromatographic test strips. These simple-to-use tools are suitable for rapid on-site screening and compatible with diverse labeling materials, including enzymes, fluorescent substances, and gold nanoparticles. Even so, such antibody-dependent systems still have undeniable limitations. Firstly, BAs belong to ultra-small haptens with simple structures and low molecular weights, which hinders the preparation of high-affinity antibodies. Secondly, conventional antibodies have complicated structures and require synergistic binding of heavy and light chains to identify targets. Thus, they are vulnerable to harsh non-physiological conditions such as organic solvents, extreme pH, and high ionic strength. This either requires tedious sample pretreatment or causes severe sensitivity loss. Although nanobodies possess outstanding stability, their production is limited by scarce experimental animal species. Additionally, their relatively low binding affinity restricts further applications in ultra-small-molecule detection.

These analytical systems have a detectable range covering common BAs, namely histamine, tyramine, and phenylethylamine, which serve as critical chemical markers for food quality and environmental safety. Among them, histamine boasts the most sophisticated detection method with favorable sensitivity and specificity due to its unique imidazole ring. In contrast, it is challenging to precisely distinguish aliphatic BAs like putrescine and cadaverine because their straight-chain alkyl structures lack distinctive functional groups. For this reason, broad-spectrum antibodies are widely adopted for the semi-quantitative screening of total BAs, acting as a powerful tool for rapid on-site freshness testing and laboratory batch screening of meat, seafood, and animal feed. These devices can process complex matrices including food, feed, and environmental specimens, although proper pretreatment remains mandatory to remove matrix interference. In summary, antibody-based recognition systems excel in the high-throughput screening of specific BAs like histamine in food supply chains, but their limited ability to discriminate structurally similar aliphatic analogues makes it difficult to meet the demand for simultaneous, accurate multi-component quantification.

### 2.2. Aptamer-Based Recognition Systems

#### 2.2.1. Screening and Specific Regulation of Nucleic Acid Aptamers

The development of aptamers commonly relies on systematic evolution of ligands by exponential enrichment (SELEX) [21], an iterative in vitro selection process designed to identify nucleic acid sequences with the highest binding affinity to target BAs from random oligonucleotide libraries containing 10^12^–10^15^ sequences. A typical DNA aptamer SELEX protocol comprises five sequential steps: oligonucleotide denaturation, single-stranded DNA-target binding, elution of bound aptamers, PCR amplification, and library recovery [22]. RNA aptamer screening requires additional reverse transcription steps post-elution and RNA oligonucleotide pool recovery via in vitro transcription after amplification. These procedures are typically repeated 8–12 times, supplemented with negative screening to obtain high-affinity aptamers. Lerga et al. [23] immobilized histamine onto magnetic beads and obtained histamine-high-affinity H_2_ aptamers (Kd = 3–34 nM) through ten rounds of SELEX screening.

Following initial screening, aptamers require optimization through a series of molecular-level modifications, with the core objectives being to maximize target specificity and minimize cross-reactivity. This process can be implemented from the screening stage, such as systematically introducing major interference factors during reverse screening. After obtaining target sequences, non-binding domains can be eliminated through truncation and mutational studies, sometimes yielding core sequences with enhanced affinity and improved specificity. To improve functional stability of aptamers in complex samples, modifications such as thio-phosphoroyl bonds or base analogs are commonly introduced into the oligonucleotide backbone to enhance their resistance to nuclease degradation.

#### 2.2.2. Aptamer-Based Recognition Mechanism

Aptamers are single-stranded DNA or RNA fragments of a defined sequence, obtained through in vitro selection, which spontaneously fold into stable three-dimensional conformations—typically stem–loop structures and G-quadruplexes—in aqueous environments, providing the structural basis for specific recognition of BAs. During molecular recognition, the target amine inserts into the pre-formed binding cavity and interacts via a combination of hydrogen bonds, electrostatic attractions, van der Waals forces, and π–π stacking. Unlike the static lock-and-key model, aptamers follow an induced-fit mechanism: target binding triggers partial or global conformational rearrangements that stabilize the complex and can be harnessed to modulate signal output, translating molecular events into measurable readouts.

However, in complex food matrices, various components exert distinct molecular interferences on different functional regions of the aptamer. High salt concentrations screen the negative charges along the phosphate backbone, thereby weakening electrostatic interactions and directly reducing binding affinity within the recognition cavity. Matrix proteins can adsorb onto the sugar–phosphate backbone or loop regions through hydrophobic patches or positively charged amino acid residues, distorting the active conformation and sterically hindering target access. Lipid molecules tend to insert into hydrophobic microdomains or the stacking planes of G-quadruplexes, disrupting the π–π stacking network essential for structural integrity. Furthermore, endogenous nucleases specifically cleave phosphodiester bonds, leading to irreversible fragmentation and loss of the intact recognition scaffold. Although aptamers offer superior chemical stability and batch-to-batch consistency compared to antibodies, these multiple molecular interferences from food components often result in significantly degraded performance in real biological environments, necessitating protective strategies such as surface modification, backbone engineering, or adequate sample pretreatment to mitigate such effects.

**Figure 2:**
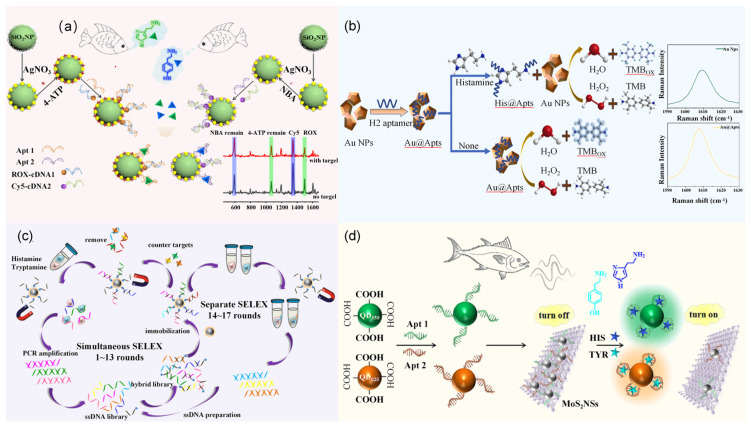
(**a**) Ratiometric SERS aptasensor using SiO_2_@Ag NPs with 4-ATP and NBA internal standards for simultaneous histamine and tyramine detection in fish. (Reprinted with permission from ref. [24]. Copyright 2023, Elsevier.) (**b**) Au NP-based histamine aptasensor for the detection of histamine in fish samples and red wine. (Reprinted with permission from ref. [25]. Copyright 2023, Elsevier.) (**c**) Simultaneous use of independent SELEX enables specific aptamer screening and subsequent detection of BAs in fish and pork. (Reprinted with permission from ref. [26]. Copyright 2023, Elsevier.) (**d**) FRET-based aptasensor employing two donors for the homogeneous and simultaneous detection of histamine and tyramine in fish samples. (Reprinted with permission from ref. [27]. Copyright 2022, Elsevier.).

In practical applications, Duan et al. [24] reported a ratiometric SERS aptasensor using SiO_2_@Ag NPs with 4-ATP and NBA internal standards for simultaneous HIS and TYR detection in fish (Figure 2a), with linear ranges of 1–100 ng/mL and detection limits of 0.2 ng/mL for HIS and 0.05 ng/mL for TYR. Wang et al. [25] established a surface-enhanced Raman scattering method based on nanozymes: aptamers adsorbed onto gold nanoparticles enhanced enzyme-like activity, while histamine-induced aptamer dissociation led to activity decline (Figure 2b). Quantification was performed using a TMB-H_2_O_2_ system, achieving a detection limit as low as 1.22 × 10^−12^ M. Duan et al. [26] employed a synchronous coupling and separation SELEX strategy to screen histamine aptamer HIS-3 (Kd = 11.62 nM) and tryptamine aptamer TRY-2 (Kd = 6.30 nM) (Figure 2c), subsequently building a colorimetric aptasensor to simultaneously quantify the two BAs in fish and meat, with respective detection limits of 1 ng/mL and 10 ng/mL. Duan et al. [27] developed an aptamer sensor based on a dual-donor FRET strategy: two aptamers were conjugated to QD530 and QD625 as donors, respectively, with molybdenum disulfide nanosheets serving as acceptors (Figure 2d). In the absence of target analytes, obvious fluorescence quenching can be observed. Histamine and tyramine are able to induce aptamer conformational changes, which further facilitate desorption and fluorescence signal recovery. The developed sensor achieves detection limits of 0.1 ng/mL and 0.25 ng/mL. The reported LODs for histamine, tyramine, and tryptamine range from 0.00014 ng/mL (nanozyme) to 1 ng/mL (colorimetric) for histamine, from 0.05 to 0.25 ng/mL for tyramine, and 10 ng/mL for tryptamine, all far below the respective safety benchmarks. For histamine, international regulatory limits are 50–200 mg/kg, whereas for tyramine and tryptamine, generally accepted advisory thresholds are around 100–200 mg/kg. Therefore, these LODs are at least three to six orders of magnitude lower, which unequivocally confirms that these biosensors can reliably identify non-compliant samples and provide ample sensitivity for early warning and routine food safety surveillance. Table 3 presents the sensing strategies based on aptamer recognition systems for BA detection in foods.

#### 2.2.3. Aptamer-Based Recognition Element Advantages, Limitations, and Application Scopes

Aptamers, as a novel class of chemical antibodies, offer significant advantages in BA detection, including a small molecular size that enables high-density immobilization on space-constrained interfaces, excellent chemical stability (particularly for DNA aptamers), high specificity and affinity comparable to antibodies, strong programmability with excellent batch-to-batch consistency, and non-immunogenicity that permits targeting a broader range of analytes. However, considerable challenges remain. The screening process is complex and costly. In complex biological matrices, performance often degrades due to nonspecific adsorption or nuclease degradation. The functional conformation and binding affinity are highly sensitive to the ionic environment. Compared with antibodies, the efficiency of generating high-affinity aptamers is lower, undermining cost effectiveness and leading to a scarcity of commercial products. Furthermore, the overall screening success rate is limited, and most studies are still confined to laboratory settings.

Currently, nucleic acid aptamer-based recognition systems for BA detection are mainly restricted to histamine, given its high risk in food poisoning, while their ability to target other BAs like tyramine and putrescine remains limited to semi-quantitative screening of total amine accumulation. Owing to their high chemical stability, flexible modification, and convenient regeneration, these aptamer sensors offer distinct advantages over conventional antibodies for complex sample analysis, maintaining strong resistance to matrix interference in diverse food specimens and environmental substrates. In terms of practical applications, this technology is highly suited for field-deployable screening and real-time freshness monitoring in seafood, meat, and fermented products, where BAs serve as key spoilage biomarkers. However, because most low-molecular-weight BAs lack complex aromatic rings or diverse functional groups to form strong hydrogen-bonding or π–π stacking networks with nucleic acid bases, screening high-affinity aptamers for a wider range of BAs remains a challenge, thereby limiting the current application scale and detectable range of this technology.

### 2.3. MIP-Based Recognition Systems

#### 2.3.1. Template Molecule Selection and Preparation Method

**Figure 3:**
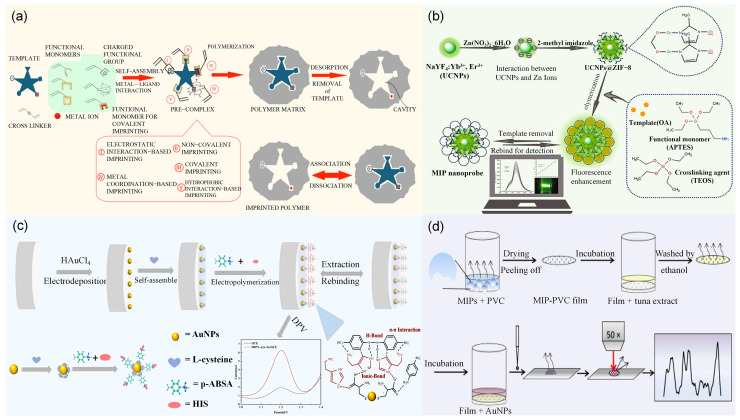
(**a**) Schematic illustration of the key steps of MIP preparation. (Reprinted with permission from ref. [36]. Copyright 2025, Elsevier.) (**b**) A molecularly imprinted fluorescent sensor was fabricated by integrating UCNPs, ZIF-8, and MIP for the detection of octopamine. (Reprinted with permission from ref. [40]. Copyright 2021, Elsevier.) (**c**) A gold nanoparticle-based molecularly imprinted electrochemical sensor for the sensitive and selective determination of histamine in alcoholic beverages. (Reprinted with permission from ref. [41]. Copyright 2015, Elsevier.) (**d**) A method employing MIPs-PVC-SERS for the quantification of histamine in canned tuna. (Reprinted with permission from ref. [42]. Copyright 2024, Elsevier.).

Molecular imprinting technology (MIT) creates artificial receptors by designing recognition sites for target compounds [34], with the core principle being the formation of specific cavities during polymerization: template molecules interact with functional monomers and crosslinkers via self-assembly, and after template removal, complementary cavities confer specific recognition [35]. The fabrication of MIPs fundamentally involves template-monomer self-assembly, crosslinking polymerization, and template removal (Figure 3a) [36]. For targeting BAs—which are typically characterized by low molecular weights, high polarities, and inherent alkalinity—the non-covalent imprinting strategy is predominantly utilized due to its operational simplicity and versatile monomer selection [37]. In this context, precise monomer selection based on host–guest chemical complementarity is paramount: acidic functional monomers like methacrylic acid (MAA) are strategically paired with the basic, often protonated amino groups of BAs, such as histamine, cadaverine, or tyramine, via strong electrostatic and hydrogen-bonding interactions. Furthermore, for BAs featuring structurally distinct motifs like imidazole rings or phenolic groups, the incorporation of alkaline or neutral co-monomers, including 4-vinylpyridine (4-VP) and APTES, or multifunctional monomer systems introduces synergistic non-covalent forces such as π–π stacking, thereby optimizing the geometric and chemical microenvironment of the imprinted cavities to significantly enhance recognition selectivity within complex matrices [38]. In a relevant study, Bongaers et al. [39] constructed an MIP-based impedance sensor by attaching MIP particles onto interdigitated electrodes; this sensing system successfully achieved an ultralow detection limit ranging from 1 to 2 nM.

#### 2.3.2. MIP-Based Recognition Mechanism

The molecular imprinting detection mechanism can be broken down into three core phases: recognition cavity generation, specific identification of target substances, and subsequent signal conversion. Throughout the polymerization process, target molecules pre-combine with functional monomers driven by non-covalent intermolecular forces like hydrogen bonds and ionic interactions, ultimately generating a densely crosslinked polymer network. After the template is stripped away, three-dimensional voids are retained in the polymer. These voids highly match the templates in terms of dimension, morphology, and functional group distribution, endowing the material with unique molecular memory capability. When detecting BAs, these target molecules are able to permeate into the structurally matched cavities again via non-covalent bonding to form stable host–guest complexes. Although such molecular binding behaviors cannot generate raw signals independently, they are capable of inducing quantifiable variations in physical parameters.

However, in real food matrices, various components interfere at the molecular level with distinct sites of the imprinted polymer. Polar pigments and phenolic compounds can penetrate the imprinted cavities and competitively displace the target amines by forming stronger hydrogen bonds or π–π interactions with the aromatic residues of functional monomers. Metal ions, such as Fe^3+^ or Cu^2+^, can coordinate with carboxylate or pyridine groups on the cavity walls, creating non-specific ionic crosslinks that distort the cavity geometry and block access. Lipids and hydrophobic proteins tend to adsorb onto the polymer surface via van der Waals forces and hydrophobic interactions, occluding the entrances to the cavities and reducing mass transfer of the target. Additionally, high salt concentrations can screen the electrostatic interactions between the target amines and the charged functional groups, weakening binding affinity within the recognition sites. These multiple, site-specific interferences often necessitate optimized washing protocols or surface modifications to preserve selectivity and recovery.

Based on the aforementioned mechanisms, Cao et al. [40] constructed a core–shell fluorescent sensor using UCNPs as the fluorescence core, ZIF-8 as the mediating layer, and surface molecularly imprinted polymer as the recognition unit for the detection of octopamine (Figure 3b), achieving a detection limit of 0.081 mg/L. Li et al. [41] fabricated a molecularly imprinted electrochemical sensor using gold nanoparticles as the conductive substrate and p-aminobenzenesulfonic acid as the functional monomer via electropolymerization for the detection of histamine in alcoholic beverages (Figure 3c), achieving a detection limit of 0.6 μM. Gao et al. [42] were the first to combine MIPs with a polyvinyl chloride membrane and surface-enhanced Raman spectroscopy (Figure 3d), establishing a method for the determination of histamine in canned tuna with a detection limit of 3 ppm. The reported LODs for histamine are 0.6 μM in the electrochemical sensor and 3 ppm in the SERS-MIP method, while the LOD for octopamine is 0.081 mg/L. All these values are far below the international regulatory limits for histamine, which range from 50 to 200 mg/kg. For octopamine, although no statutory maximum level exists, the obtained sensitivity is still orders of magnitude lower than the generally recommended safety thresholds for BAs in foods (typically 100–200 mg/kg). Hence, these molecularly imprinted sensors can reliably identify non-compliant samples and offer ample sensitivity for routine food safety surveillance. Table 4 presents the sensing strategies based on MIP recognition systems for BA detection in foods.

#### 2.3.3. MIP-Based Recognition Element Advantages, Limitations, and Application Scopes

Compared with traditional detection methods, MIPs, as synthetic recognition elements, exhibit excellent chemical stability and maintain good performance under high temperature, strong acid, strong alkali, and organic solvent conditions. To some extent, they address the issues of insufficient sensitivity and high operational complexity associated with conventional methods. Moreover, MIPs do not rely on the biological immune system, allowing, in principle, the preparation of specific recognition materials for any BAs. They are particularly suitable for selective enrichment and detection of structurally similar analogues using a “dummy template” strategy. However, this technology still has notable limitations, mainly the limited variety of functional monomers and insufficient diversity of imprinting methods, leading to heterogeneous binding sites and room for improvement in affinity.

MIPs have been widely applied for the detection of diverse common BAs, such as histamine, tyramine, putrescine, cadaverine, phenylethylamine, and spermine, which serve as critical indicators of freshness and spoilage. Of all available imprinted materials, histamine-tailored polymers have attracted the greatest research attention due to the high toxicity of this specific amine, achieving documented detection limits spanning from nanomolar to micromolar scales. Unlike fragile biological antibodies, MIPs tolerate rigorous sample pretreatment conditions and maintain stable recognition of the aliphatic or aromatic amino structures of BAs within complicated matrices, such as food specimens and environmental substrates. Serving as robust adsorbents for solid-phase extraction, these polymers are commonly coupled with HPLC or liquid chromatography–mass spectrometry (LC-MS) for the efficient enrichment and precise quantification of trace BAs. Beyond laboratory-bound analysis, the chemical stability of MIPs allows them to be integrated into electrochemical and optical sensing devices for rapid, on-site field detection during food processing and storage. Despite prominent chemical inertness and excellent anti-interference capacity in these practical analytical scenarios, MIPs still lag behind antibody-based receptors in molecular recognition accuracy.

### 2.4. Enzyme-Based Recognition Systems

#### 2.4.1. Natural Enzymes for BAs

Natural enzymes serve as vital biorecognition components, which are capable of achieving highly specific detection toward BAs. This section focuses on research advancements in BA-degrading enzymes such as amine dehydrogenases (AmDHs), monoamine oxidases (MAOs), diamine oxidases (DAOs), and multicoenzyme oxidases (MCOs).

AmDHs catalyze the dehydrogenation of amines to produce corresponding aldehydes and ammonia, with cofactors typically being tryptophan tryptophan quinone (TTQ) or cysteine tryptophan quinone (CTQ). The majority of AmDH variants are modified from structurally homologous natural enzymes via protein engineering techniques [54]. Franklin et al. [55] successfully generated an L-AmDH mutant by inserting two amino acid mutations within the substrate-binding pocket of LeuDH. Such structural alterations remodeled its oxidative deamination pathway and enhanced substrate inhibition effects, which renders this engineered enzyme more adaptable for industrial utilization.

MAO is a class of enzymes that trigger the oxidation reaction of monoamines. It contains two isoforms, namely MAO-A and MAO-B; these two isoenzymes are encoded by distinct genes and exhibit divergent performances in inhibitor susceptibility and substrate selectivity [56]. Deng et al. [57] conducted heterologous expression of the MAO5 enzyme in various expression systems. Their experimental outcomes uncovered obvious activity discrepancies of this enzyme in different strains. In particular, the MAO5-ZMU6 strain possessed the optimal enzymatic activity under ambient conditions of pH 7.5 and 37 °C.

DAO, also known as histamine oxidase, is a homodimeric enzyme composed of 751 amino acid residues, with a protein-derived cofactor TPQ [58]. Kettner et al. [59] utilized the CRISPR-Cas9 system to integrate the DAO homologous recombinant construct (DAO-1) from *P. oleifera* PO1f into its genome, resulting in functional DAO activity 93-fold higher than that of the native enzyme. This recombinant enzyme can act on various aniline compounds.

**Figure 4:**
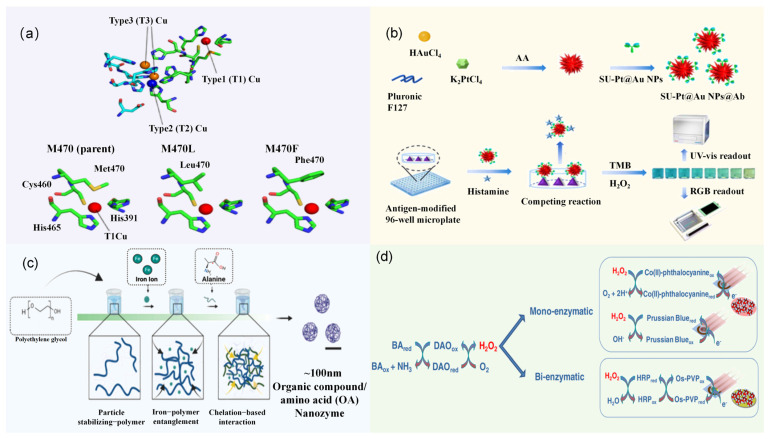
(**a**) The copper atoms of MCO are classified as type 1, type 2, and type 3 copper based on their electronic and magnetic properties. (Reprinted with permission from ref. [60]. Copyright 2021, Springer Nature.) (**b**) A dual-mode colorimetric strategy for histamine sensing, employing SU-Pt@Au NPs as a peroxidase-mimicking nanozyme. (Reprinted with permission from ref. [62]. Copyright 2024, Elsevier.) (**c**) A customized “single-chelation/polymer-entanglement” procedure was employed to synthesize an OA nanozyme for histamine sensing. (Reprinted with permission from ref. [63]. Copyright 2025, Elsevier.) (**d**) An electrochemical enzyme sensor array for the detection of histamine, putrescine, and cadaverine using magnetic beads as the immobilization support. (Reprinted with permission from ref. [66]. Copyright 2016, Springer Nature.)

The four copper atoms present in MCOs are assigned to type 1, type 2, or type 3 (abbreviated as T1Cu, T2Cu, and T3Cu) on the grounds of their electronic and magnetic characteristics. [60] (Figure 4a). Zeng et al. [61] isolated a novel copA gene from the plant bacterium MK-8, expressed MCO through overexpression in Escherichia coli, and successfully enhanced catalytic efficiency by mutating Cys495 to Asp, His, and Met via directed mutagenesis.

#### 2.4.2. Nanozymes: Advantages over Natural Enzymes

Nanoenzymes represent artificially engineered nanomaterials with catalytic functions, and their catalytic capacities are predominantly determined by surface physicochemical characteristics. Rational structural optimization enables these nanomaterials to acquire excellent substrate specificity toward target molecules. In comparison with natural biocatalysts, nanoenzymes feature affordable manufacturing costs, facile synthetic routes, improved catalytic efficiency, and superior stability under extreme environments, effectively mitigating enzyme deactivation risks. Owing to these favorable traits, nanoenzymes have garnered extensive application potential across biosensing, agricultural monitoring, and food safety analysis. In recent research, Zhang et al. [62] fabricated sea urchin-like Pt@Au nanozymes functioning as peroxidase mimics to construct a combined colorimetric and smartphone-based dual-mode immunoassay system (Figure 4b). This sensing platform realized high-sensitivity histamine quantification in Pacific saury, crab, and pork samples, with a low detection limit of 0.15 ng/mL. Furthermore, Lee and Kamruzzaman [63] adopted a facile single-chelation and polymer-entanglement strategy to synthesize an eco-friendly organic nanozyme (OA nanozyme) using L-alanine and polyethylene glycol (Figure 4c). The resulting OA nanozyme possesses intrinsic peroxidase-mimicking activity, and its catalytic behavior can be regulated through electrostatic interactions. This property facilitates rapid colorimetric histamine detection, achieving a detection limit as low as 21.37 pg/mL.

#### 2.4.3. Enzyme-Based Recognition Mechanism

Enzyme-modified biosensors achieve BA detection largely by relying on specific oxidative reactions. Such biochemical processes are catalyzed by a variety of amine oxidase family enzymes, typically including diamine oxidase, monoamine oxidase, polyamine oxidase, and histamine dehydrogenase. These enzymes efficiently catalyze the oxidative deamination of target BAs, generating corresponding aldehydes, ammonia, and hydrogen peroxide (H_2_O_2_). Signal transduction follows two main pathways: direct electrochemical oxidation or reduction of H_2_O_2_ at the working electrode, generating a current linearly proportional to amine concentration, or horseradish peroxidase (HRP)-mediated chromogenic or electrochemical amplification using the enzymatically produced H_2_O_2_.

At the molecular interference level, different food components selectively attack specific functional sites of the enzyme sensor. Reducing substances such as ascorbic acid and glutathione do not directly act on the substrate-binding pocket but instead scavenge the enzymatically generated H_2_O_2_, thereby interrupting the signal amplification chain and weakening the detection response. High concentrations of salt ions can shield the electrostatic attractions between charged residues near the active center (such as lysine and arginine) and the amino groups of the substrate or alter the ionic strength to affect electron transfer efficiency between cofactors and the substrate, thus reducing the catalytic rate. pH fluctuations modify the protonation state of key active-site amino acids, disturbing the hydrogen-bond network with the substrate and the redox potential of cofactors, thereby compromising enzymatic activity. Furthermore, lipids and proteins in the food matrix can non-specifically deposit onto the electrode surface through hydrophobic interactions or electrostatic adsorption, forming a physical insulating layer that not only impedes electron transfer but also restricts substrate diffusion toward the enzyme active center.

In practical application research, Torre et al. [64] developed a time-efficient amperometric sensor in 2020. They immobilized diamine oxidase extracted from porcine kidneys onto screen-printed carbon electrodes for hydrogen peroxide detection, which could output stable analytical signals within 60 s. When applied to histamine quantification in buffer solutions and fish extracts, this sensor achieved an LOD of approximately 1 mg/L, alongside a linear detection range spanning 1 to 100 mg/L. Henao-Escobar et al. [65] constructed a dual-enzyme sensor array employing histamine dehydrogenase and putrescine oxidase, which allowed for the simultaneous discrimination and measurement of these two BAs in samples. Leonardo and Campás [66] used magnetic beads to immobilize diamine oxidase and constructed an electrochemical enzyme sensor array for the detection of histamine, putrescine, and cadaverine (Figure 4d), with detection limits of 4.50 μM for histamine, 0.90 μM for putrescine, and 0.47 μM for cadaverine. The reported LODs are 1 mg/L for histamine and 0.50 mg/L (4.50 μM), 0.079 mg/L (0.90 μM) for putrescine, and 0.048 mg/L (0.47 μM) for cadaverine. For histamine, international regulatory maximum levels are 50–200 mg/kg, meaning these LODs are at least 50-fold lower. For putrescine and cadaverine, although no statutory limits exist, the obtained sensitivities are also several orders of magnitude below the generally recommended total biogenic amine thresholds (100–200 mg/kg). Thus, these enzyme sensors reliably discriminate compliant from non-compliant samples and offer adequate sensitivity for early spoilage detection and routine food safety monitoring. Table 5 presents the sensing strategies based on enzyme recognition systems for BA detection in foods.

#### 2.4.4. Enzyme-Based Recognition Element Advantages, Limitations, and Application Scopes

Enzyme-based recognition systems achieve precise identification and signal amplification of BAs through their “lock-and-key” specific binding mechanism and efficient catalysis. Enzymatic sensors possess multiple superior properties: rapid detection speed, simple operation, acceptable regenerability after immobilization, and favorable substrate selectivity, alongside high sensitivity and flexible detection modes. Nevertheless, these biological recognition elements exhibit noticeable limitations. Enzymatic molecules are structurally unstable and prone to inactivation under extreme temperature, unsuitable pH values, organic solvents, or fluctuating ionic strength. Additionally, individual enzymes generally feature a narrow recognition spectrum, which hinders the simultaneous identification of diverse BAs. Reductive compounds present in intricate food matrices also create detection interference. Furthermore, high production costs, narrow linear ranges, and susceptibility to environmental changes constrain the long-term detection reliability of enzyme-based sensors in complex harsh conditions.

This enzyme system is uniquely suited for the dynamic monitoring of total BAs and on-site rapid safety assessment, focusing primarily on food quality evaluation and environmental monitoring. By interacting with the shared aliphatic or aromatic amino structures of common BAs—such as putrescine, cadaverine, histamine, tyramine, spermine, and spermidine—this approach provides an estimation of total amine accumulation, which serves as a critical index for food spoilage. The system is highly compatible with liquid samples or those obtained through simple extraction, making it ideal for high-throughput, field-deployable testing of seafood, meat, and fermented products. While the varying alkyl chain lengths of these specific BAs influence their individual reaction kinetics, complex matrices characterized by high fat, high protein, or dark coloration simply require straightforward pretreatments like dilution, filtration, or decolorization to eliminate matrix interference and ensure reliable on-site quantification.

### 2.5. Peptide-Based Recognition Systems

#### 2.5.1. Screening and Design Strategies for Peptide Sequences

Peptides consist of 20 natural amino acids, and variable sequence arrangements endow them with diverse structures and biological functions, making them competitive recognition elements. Phage display represents the most mainstream technique for peptide screening. This method constructs random peptide libraries with a diversity of over 10^9^ unique sequences. After multiple rounds of affinity panning, high-affinity peptide sequences can be screened out for specific BAs, including histamine, tyramine, and cadaverine. For example, Hafize Öz et al. [72] used a modified 12-mer library (excluding histidine binders) to screen histamine-specific peptides HBF10 and HBF26 with no histidine cross-reactivity and binding constants of 2.56 × 10^4^ M^−1^ and 8.94 × 10^4^ M^−1^. Beyond phage display, mass spectrometry directly screens binding peptides from libraries and identifies BA recognition sequences within natural antimicrobial peptide (AMP) libraries.

#### 2.5.2. Peptide-Based Recognition Mechanism

Peptide recognition of BAs relies on sequence-specific chemical properties and three-dimensional conformations. At physiological pH, protonated BAs carry a positive charge, making electrostatic interactions the primary binding force. Peptides rich in acidic amino acids, such as aspartic acid and glutamic acid, form negatively charged regions to capture these cationic amines. Hydrogen-bond networks further enhance specificity, with amine groups serving as both donors and acceptors to interact with amide bonds and hydroxyl side chains of serine, threonine, and tyrosine. For aromatic BAs like tyramine and histamine, hydrophobic interactions and π–π stacking become critical. Incorporating aromatic residues like tryptophan, tyrosine, and phenylalanine promotes stable complex formation through aromatic stacking. Additionally, hydrophobic regions in the peptide backbone minimize water interference at the binding interface.

In complex food matrices, different components exert molecular interferences on distinct functional sites of the peptide. High concentrations of salt ions screen the electrostatic attraction, directly weakening the binding force between the negatively charged regions and the cationic amine groups. Extreme pH conditions alter the protonation state of acidic/basic residues, disrupting the electrostatic and hydrogen-bond networks and compromising the charge complementarity of the binding cavity. Endogenous proteases hydrolyze peptide bonds, cleaving the backbone structure and causing degradation of the recognition unit. Furthermore, lipids and hydrophobic small molecules can insert into the hydrophobic regions of the peptide through hydrophobic interactions, interfering with π–π stacking between aromatic residues, while pigment molecules may compete for hydrogen-bond sites, increasing non-specific binding. Although cyclization modifications and multivalent design can enhance conformational stability and improve affinity, the aforementioned multiple interferences may still significantly reduce the binding activity of peptides in real food systems, necessitating complementary strategies such as rational sample pretreatment or peptide engineering (e.g., incorporation of non-natural amino acids) to ensure detection reliability. Table 6 presents the sensing strategies based on peptide recognition systems for BA detection in foods.

#### 2.5.3. Peptide-Based Recognition Element Advantages, Limitations, and Application Scopes

Chemical modification, cyclization treatment, and multivalent structural design are proven effective strategies to substantially improve the recognition performance of peptides toward BAs. The introduction of unnatural amino acids can enhance the protease resistance of peptides while maintaining their conformational stability. Cyclization driven by disulfide linkages or amide bonds constrains molecular flexibility and lowers entropy loss, further boosting the binding affinity between peptides and targets. Furthermore, multivalent peptide immobilization on skeletons or nanoparticles generates favorable synergistic effects, which can elevate binding constants by one to two orders of magnitude. Despite such prominent merits, peptide-based sensing still encounters practical limitations in real food matrices. Complex food ingredients containing abundant salts, miscellaneous proteins, and extreme pH conditions may destroy peptide structures and weaken intermolecular binding interactions. Although peptides exhibit better stability compared with antibodies, they are still vulnerable to proteolytic degradation, thereby undermining long-term detection stability. In addition, high structural homology across different BA molecules poses difficulties for accurate differentiation and specific identification.

### 2.6. Others

Traditional recognition elements, including enzymes, antibodies, aptamers, and MIPs, have long dominated BA sensing research, accompanied by the continuous emergence of innovative recognition strategies. Metal–Organic Frameworks (MOFs) are defined as porous crystalline materials that possess large specific surface areas and customizable pore architectures. These materials are capable of generating multiple intermolecular forces, such as coordination bonding, π–π stacking, and electrostatic adsorption, thus achieving efficient molecular capture. Within sensing systems, MOFs can display inherent fluorescence properties, demonstrate nanozyme activity to generate colorimetric and fluorescent signals, or act as electrode modification coatings for the fabrication of high-sensitivity electrochemical sensors.

Covalent organic frameworks (COFs) allow for controllable surface functionalization. The grafting of amino, carboxyl, or hydroxyl functional groups facilitates hydrogen bonding, π–π stacking, and electrostatic interactions, thereby greatly improving their binding strength and molecular recognition capability. Owing to their large specific surface area and rigid structure, COFs can serve as recognition media for selective enrichment and sensing. The rational regulation of pore size and channel geometry can further optimize molecular binding efficiency, accelerating their scalable implementation in recognition-related detection scenarios.

Whole-cell biosensors adopt genetically modified bacteria, yeast, or mammalian cells as biological sensing units. By combining BA-responsive components with reporter genes, these biosensors convert analyte concentrations into measurable signals. Such systems can mimic biological behaviors under near-physiological environments and support the simultaneous monitoring of multiple metabolic pathways. Nevertheless, their inherent drawbacks, including slow response kinetics, strict culture requirements, metabolism-dependent signals, and bulky supporting equipment, restrict their practical on-site deployment.

## 3. Signal Transduction Strategies in Rapid Detection of BAs

### 3.1. Photochemical Analysis Method

#### 3.1.1. Colorimetric Methods

The colorimetric method achieves qualitative, semi-quantitative, or quantitative analysis of target substances by analyzing the color intensity of colored solution samples [77]. The enzymatic reaction system, the earliest classical method for bioamine colorimetric analysis, centers on specific recognition enzymes like tyrosinase and diamine oxidase combined with horseradish peroxidase to form a cascade system, offering rapid response and strong recognition capability. Nevertheless, enzymatic performance is extremely susceptible to temperature fluctuations, pH variation, and organic solvents. Such vulnerability causes inherent limitations, including easy inactivation, rigorous storage conditions, and poor recyclability, thereby hindering their scalable deployment in complicated on-site detection environments. Saedan et al. [78] extracted cellulose from recycled newspaper and immobilized diamine oxidase and a pH indicator as recognition elements to construct a colorimetric sensor for detecting cadaverine, putrescine, and histamine, successfully applying it to monitor pork spoilage.

To address enzymatic instability, colorimetric detection technology based on MIPs has emerged accordingly. Compared to enzymatic systems, it is more stable, reproducible, cheaper, and field-suitable but has poor specificity, cross-reactivity, weak interference resistance, and template leakage causing false positives. To address the inherent limitations of molecular imprinting systems in target recognition specificity, highly specific antibody-or nucleic acid aptamer-based biorecognition colorimetric systems have gradually become a research focus. This leverages high specificity/affinity, overcoming cross-reactivity with excellent selectivity and interference resistance. However, antibody instability and cumbersome multi-step procedures requiring skilled personnel limit on-site screening efficiency.

In the colorimetric detection of BAs, commonly used materials primarily include organic dyes, metal nanoparticles, natural pigments, and functional composite systems. Natural pigments (such as anthocyanins) and surface-modified metal nanoparticles can directly serve as pH-sensitive chromogenic materials, working synergistically with enzymes or molecularly imprinted elements to enable low-cost detection. For example, Huang et al. [79] used an anthocyanin/pectin-MIL-100(Fe) sensor for amine gases, where the MOF enhances adsorption via chemisorption and intraparticle diffusion, greatly improving sensitivity to volatile BAs. The colorimetric detection of BAs often relies on non-nanomaterials as well, such as organic dyes, polymers, and redox indicators. In one illustrative study, Lyu et al. [80] established an azo-derived colorimetric assay for histamine quantification. Gradual color transition from yellow to orange-red and ultimately pink could be observed with increasing histamine concentrations. This method exhibited a robust linear correlation with a coefficient of determination (R^2^ = 0.9926), achieving a detection limit lower than 20 μg/mL.

#### 3.1.2. Fluorescence Method

Fluorescent sensing has emerged as a prevalent signal readout for visual detection. This technique boasts high detection sensitivity, rapid response speed, negligible background noise, and convenient automated operation [81]. The earliest enzyme-based fluorescence systems typically combined oxidases with fluorescent substrates and quantum dots, where enzymatic catalysis of bioamine oxidation generates hydrogen peroxide to trigger the fluorescence response. For example, the dual-target fluorescent probe HDXM developed by Yang et al. [82] utilizes enzymatic catalysis and dual recognition technology, achieving a bioamine response within just 3 s with a detection limit as low as 6.89 μM. However, enzymes are environmentally sensitive, costly, batch-unstable, and non-reusable, limiting long-term on-site detection. To address the stability bottleneck of enzyme-based systems, molecularly imprinted polymer (MIP)-based fluorescent systems were developed. Compared to enzyme-based systems, polymer backbones offer environmental robustness, controllable production, reuse, and lower costs. However, spatial structures and weak interactions cause cross-recognition of similar bioamine homologs. Additionally, aqueous nanoparticle aggregation raises fluorescence and reduces stability, lowering specificity vs. bioaffinity.

Antibody/aptamer bioaffinity fluorescence systems overcome enzyme stability and MIP specificity limits as next-generation core tools. Aptamers outperform MIPs in specificity for similar compounds, with robustness and dispersibility avoiding aggregation issues. Antibodies are costly, slow, hard to screen for small BAs, and unstable, while aptamers have tedious screening and low-quality sequences. Both risk affinity loss in complex matrices. Building on this, lanthanide MOFs are hotspots for dual recognition/luminescence, with research trending toward multi-technology integration.

#### 3.1.3. Chemiluminescent Mode

Chemiluminescent sensors are developed based on the emission of light (ultraviolet, visible, or infrared), which is created due to chemical reactions [83]. The first practical enzyme-based chemiluminescence system enables rapid, specific, low-cost, and user-friendly on-site visual detection. For example, Omanovic-Miklicanin and Val zacchi [84] fabricated chemiluminescence sensors using putrescine oxidase and diamine oxidase to detect putrescine in fish and meat, with respective detection limits of 0.8 and 1.3 mg/L. However, enzymes are vulnerable to environmental conditions, costly, unstable, and non-reusable, limiting their use in high-frequency on-site food detection. To overcome the poor tolerance and high costs of enzyme-based systems while retaining specificity, antibody/aptamer-based immune-affinity chemiluminescence systems have emerged. These systems outperform enzymatic ones in selectivity, reduce analog cross-interference, and broaden application scope. Xu et al. [85] generated mouse mAbs against histamine and constructed an ELISA and CLEIA for its detection, with detection limits of 89.0 ng/mL and 73.4 ng/mL. However, these systems involve multi-step cycles, costly unstable antibodies, and high-cost aptamers with difficult screening, making them still unable to resolve the fundamental limits of biorecognition elements.

MIP chemiluminescence overcomes the environmental stability limits of antibody and nucleic acid recognition. Compared to antibodies and enzymes, these systems offer environmental robustness, reusability, and low cost, overcoming inactivation and non-reusability. However, relying on spatial and weak interactions, these systems cross-react with similar BAs, and their specificity and catalytic efficiency are below biological affinity elements and natural enzymes, limiting sensitivity. Gold nanoparticles and carbon dots enhance chemiluminescence signal intensity, improving efficiency and sensitivity. With high surface area and tunable sites, MOFs integrate enrichment and specific amine detection, overcoming poor specificity and enrichment. Detection of BAs using chemiluminescence has developed in response to core analytical needs and has expanded to enable rapid on-site food safety monitoring.

### 3.2. Electrochemical Analysis Methods

Electrochemical transduction enables quantification by measuring changes in current, potential, or impedance, with common methods including voltammetry, electrochemical impedance spectroscopy (EIS), and amperometry (AMP).

#### 3.2.1. Voltammetric Analysis

Early voltammetric enzymatic sensors immobilized diamine oxidase and monoamine oxidase on electrode surfaces modified with Nafion or chitosan membranes. Thanks to their high catalytic activity and substrate specificity, these enzymes oxidize BAs into electroactive products, amplifying electrical signals for highly sensitive and selective detection. However, enzymes are sensitive to temperature, pH, and ionic strength, which can easily lead to protein inactivation. The immobilization process also tends to reduce enzymatic activity, resulting in poor operational stability and high fabrication costs. These limitations significantly restrict their large-scale use in on-site testing. To overcome enzyme instability while maintaining excellent specificity, antibody-based voltammetric sensors have been developed. Antibodies are immobilized on gold or carbon electrodes through oriented adsorption or covalent crosslinking, enabling precise immunoaffinity recognition. While these immunosensors offer high selectivity and detection accuracy, they are still affected by protein denaturation, strict storage requirements, complex immobilization procedures, and high costs, which limit their practical, low-cost application in field testing.

Beyond systems based on specificity, enzyme-free materials such as carbon nanomaterials combined with Au, Pt, or transition metal oxides can catalyze BA oxidation at lower overpotentials. For instance, Lourenço et al. [86] developed a NiPC-modified carbon paste electrode for square wave voltammetric detection of tryptamine and histamine in wines, achieving enhanced current responses and detection limits of 0.168 ng/mL and 0.038 µg/mL at pH 8.5. By strategically combining recognition elements with electrode materials, voltammetry enables efficient detection of a variety of BAs.

#### 3.2.2. EIS

EIS is a key technique that measures impedance, expressed in ohms, within a circuit. In detecting BAs, gold or glassy carbon electrodes provide recognition and signal amplification through interfacial modifications, while MIPs create surface cavities that increase charge transfer resistance by physically blocking electron flow. Different imprinting strategies, however, can lead to opposite impedance responses. For instance, Venkatesh et al. [87] developed a histamine sensor using a metal–organic polymer (MIPs with methacrylic acid as the functional monomer) combined with EIS. In this system, a higher histamine level strengthens ionic bonding, which reduces charge transfer resistance and overall impedance.

Aptamers immobilized on gold via thiol self-assembled monolayers (SAMs) restrict probe diffusion when binding amines, while antibodies change impedance by forming an inert immune complex layer, often using protein A or crosslinking. To improve sensitivity, materials such as gold nanoparticles, carbon nanotubes, graphene, or MOFs are commonly added as signal amplification layers, and substances like Nafion, chitosan, or polyethylene glycol help reduce nonspecific adsorption. Additionally, diamine oxidase-based impedimetric sensors can detect BAs through enzymatic reactions that alter the local microenvironment. The combined effects of these strategies allow EIS to selectively and quantitatively measure BAs even in complex sample matrices.

#### 3.2.3. AMP

AMP relies on recording current variations at a fixed applied potential to achieve the quantification of histamine and other BAs. Various recognition systems differ noticeably in detection sensitivity and selectivity, each possessing unique strengths and inherent drawbacks. Enzymatic sensors work by capturing hydrogen peroxide generated from enzymatic reactions and thus exhibit excellent selectivity. Such sensors commonly adapt Prussian blue-modified electrodes combined with nanomaterials to amplify response signals and deliver fast detection output, yet enzymes themselves suffer from weak environmental stability. Aptamer-based sensors regulate current signals via the target-triggered displacement of electroactive labels. Compared with enzymatic sensors, they present better stability, though their binding affinity toward small-molecule targets is relatively limited. MIP-based sensors block electron transmission through internal imprinted cavities. They show strong anti-interference ability in complex sample matrices, but it is essential to effectively suppress nonspecific adsorption effects during application. Antibody-based immunosensors utilize immunoaffinity binding and enzymatic labels to realize current signal amplification, which endows them with outstanding selectivity, while the whole detection workflow tends to be more cumbersome. In general, these sensing platforms differ greatly in response rate, stability level, and operational difficulty, so the selection should be tailored to the actual demands of practical detection scenarios.

Platinum and nickel electrodes are also applicable to direct enzyme-free oxidation reactions. Among them, nickel can form a catalytic layer under alkaline conditions, yet both electrode types are susceptible to surface contamination. Gajjala and Palathedath [88] deposited a Cu@Pd core–shell nanostructure onto the sensor surface. These nanostructures simultaneously serve as catalytic substances and signal stabilizers. The constructed sensor system can realize histamine oxidation at a potential of +0.55 V and generate current signals that change proportionally with the target concentration. It effectively weakens the interference caused by ascorbic acid and uric acid and avoids the degradation of catalytic materials, thereby supporting stable and credible on-site detection for complicated food sample matrices.

## 4. Integrated and On-Site Detection

### 4.1. On-Site Detection by Portable Devices

Lightweight and convenient, portable devices dominate POCT, generating numerous tools for rapid on-site food safety testing. These encompass standard paper-based devices alongside emerging smart labels and microfluidic chips that enhance both intelligence and efficiency.

#### 4.1.1. Test Strips

Lateral flow immunoassay strips have emerged as a mainstream field detection platform, leveraging capillary chromatography and solid-phase competitive immunoassay mechanisms. These tools offer simple operation, low preparation costs, and no professional instruments, with easily user-interpretable results. Nevertheless, significant limitations persist. Detection yields only semi-quantitative results with relatively poor sensitivity, and complex food sample components frequently cause interference. Current research targets performance enhancement and intelligent upgrades through multiple strategies. Current research efforts therefore target both performance enhancement and intelligent system upgrades, with a parallel emphasis on optimizing time efficiency for practical deployment.

Conventional colloidal gold-based LFIAs, including sample homogenization and dilution, typically require a total assay time of 10–15 min. To enhance sensitivity, the introduction of fluorescent nanoparticulate labels increases the total assay duration to 20–25 min, owing to slower antigen–antibody binding kinetics. Multiplexed detection configurations, designed for simultaneous identification of multiple targets, necessitate reduced flow rates on the nitrocellulose membrane to avoid cross-reactivity, extending the single-run time to 20–30 min. Furthermore, smartphone-based image analysis, performed after the strip has fully developed (typically at the completion of lateral flow), requires only 1–3 min of algorithmic processing to convert T/C line intensity ratios into quantitative readouts, thereby overcoming the limitation of semi-quantitative analysis with minimal time overhead. The overall workflow efficiency is substantially superior to that of laboratory reference methods such as the enzyme-linked immunosorbent assay (1.5–2 h) or quantitative PCR (1–2 h). In summary, the entire procedure—including pretreatment and necessary signal acquisition—can be completed within 15–30 min, a timeframe that fully meets the throughput requirements for food safety screening at collection points and agricultural markets.

**Figure 5:**
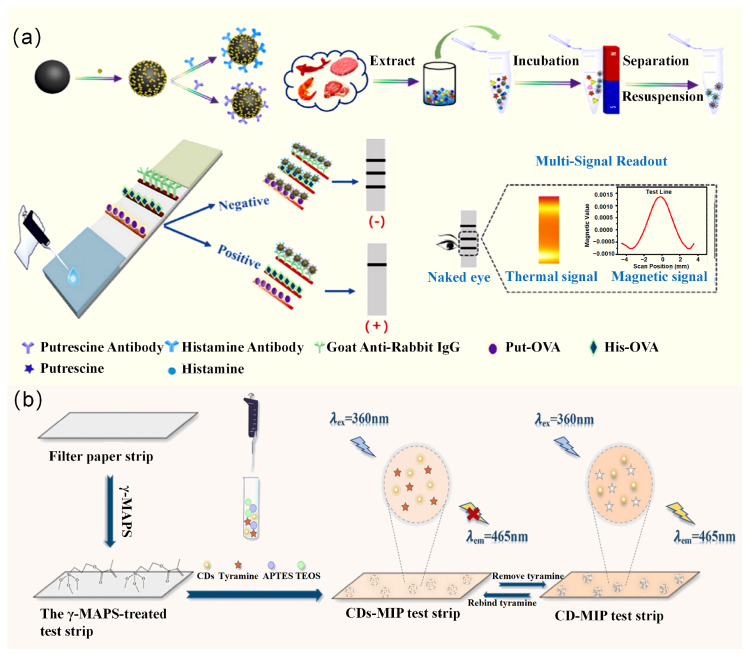
(**a**) A multi-signal readout LFIA platform based on Fe_3_O_4_@Au for the detection of putrescine and histamine. (Reprinted with permission from ref. [90]. Copyright 2023, Elsevier.) (**b**) A fluorescent sensing test paper based on CDs-MIPs developed via sol–gel in situ synthesis for the detection of tyramine in vinegar. (Reprinted with permission from ref. [92]. Copyright 2021, Elsevier.).

In this context, Mairal-Lerga et al. [89] developed a lateral flow assay (LFA) by optimizing the truncation of the H2 histamine aptamer and combining it with a partially complementary capture DNA probe. The method was successfully applied for rapid detection of histamine in canned tuna and sardines, achieving detection limits of 22.8 nM for fish samples. Yang et al. [90] developed a “three-in-one” LFIA platform based on Fe_3_O_4_@Au nanocomposites, integrating magnetic enrichment, colorimetric detection (Figure 5a), and photothermal signal output functions. The triple readout signals enabled quantitative detection of putrescine and histamine with detection limits as low as 0.17 ng/mL and 0.31 ng/mL. Li et al. [91] developed a two-site chemical recognition test strip based on polydopamine-coated gold nanoparticles. The strip utilizes the reaction between carboxyl groups and the amino group of histamine, along with the specific binding of NTA-Cu^2+^ chelates to its imidazole group, enabling rapid colorimetric detection of the target analyte in samples such as tuna and salmon, with a detection limit of 1.16 μg/mL. Qiao et al. [92] developed a fluorescent test strip combining carbon dots with CDs-MIPs for rapid on-site detection of tyramine (Figure 5b), achieving an imprinting factor of 4.96. Fluorescence emission showed linear quenching over 0.5–10.0 mg/L, with a detection limit of 0.059 mg/L.

The LODs reported in these lateral flow and fluorescent strip assays are 22.8 nM for histamine, 0.31 ng/mL for histamine and 0.17 ng/mL for putrescine, 1.16 μg/mL for histamine, and 0.059 mg/L for tyramine. All these values are substantially below the respective safety benchmarks. For histamine, international regulatory limits range from 50 to 200 mg/kg. For putrescine and tyramine, the generally recommended advisory levels are 100–200 mg/kg, although no statutory limits are universally established. Thus, these strip-based sensors reliably identify non-compliant samples and provide ample sensitivity for on-site screening. From a holistic perspective, existing detection approaches reveal an obvious developmental trend. Lateral flow test strip platforms are gradually moving toward higher intelligence, improved sensitivity, and diversified functionality. In practical testing, it is necessary to reasonably select recognition elements such as aptamers, antibodies, chemical chelates, and MIPs. Meanwhile, signal output modes including colorimetric, photothermal, and fluorescent types should also be properly matched according to actual sample composition and sensitivity requirements. Subsequent research can devote more efforts to simplifying complex multi-step processes represented by Yang’s magnetic enrichment strategy. It is also important to further strengthen the real-time quantitative detection capacity to better meet the on-site POCT application demands.

#### 4.1.2. Non-Invasive Smart Tags

Non-invasive smart tags represent a novel technology for directly monitoring the freshness of packaged foods. These tags work without contacting the food or opening the packaging, as they selectively react with volatile BAs released during spoilage. This reaction triggers measurable changes in fluorescence or color, allowing for contactless, real-time assessment of food quality. Typically, smart tags respond within 15–30 min of initial exposure to the headspace atmosphere inside the package, generating a distinct optical signal readable by a handheld device or a smartphone camera. Thereafter, the tags automatically update the readings at 5–10 min intervals, providing continuous freshness profiles throughout the entire storage period. The technique requires neither sample pretreatment nor addition of any reagents; the only manual operation is the initial attachment of the tag (taking less than 1 min), rendering hands-on time virtually negligible.

In contrast to conventional methods that rely on destructive sampling, yield delayed results (1–3 h for laboratory assays), and fail to track freshness dynamically, smart tags deliver actionable data within 30 min and enable ongoing monitoring, thereby eliminating repeated sampling and offering real-time warning of quality deterioration without extra time costs. Their overall time efficiency is substantially superior to that of conventional end-point detection methods, making them particularly suitable for field applications that demand rapid and frequent assessments.

Recently, Wang et al. [93] developed a ratiometric fluorescent probe by coupling FITC to an NH_2_-rich EuMOF. This probe shows a dual-emission response to BAs: as the concentration of amines increases, the green fluorescence from FITC becomes stronger, while the red emission from Eu^3+^ diminishes. This shift in emission causes the observed color to change from orange-red to green, allowing for simple detection with the naked eye. Specifically, for histamine, the probe demonstrates a linear detection range of 2.78–41.67 mg·L^−1^ and a detection limit of 1.11 mg·L^−1^. In addition to histamine, the probe is also responsive to several other common BAs, making it a versatile tool for monitoring food spoilage. Liu et al. [94] designed a cost-effective color-changing smart label for freshness monitoring using a hybrid deposition technique combining fluorescein and carbon quantum dots (RQDs), achieving a breakthrough in the visual detection of BAs.

Wang’s histamine LOD of 1.11 mg/L is 45-fold below the strictest international limit (50 mg/kg). For other BAs, though lacking statutory limits, recommended total amine thresholds (100–200 mg/kg) are far above this sensitivity. Liu’s label responds at early spoilage levels, below hazardous thresholds. Both strategies reliably identify unsafe or non-compliant samples and are suitable for rapid visual screening. In summary, both strategies enable naked-eye detection of BAs, but each addresses different practical challenges. Wang’s ratiometric sensor overcomes the instability often seen in single-emission probes by incorporating an internal self-calibration mechanism, which enhances quantification accuracy and reduces the influence of environmental factor. On the other hand, Liu’s smart label prioritizes low cost and compatibility with packaging, accepting a modest reduction in analytical precision in exchange for easier scalability and real-world application. While Wang’s approach offers superior quantitative stability and clearer color differentiation, Liu’s method shines in mass production and on-site deployment. Ultimately, the choice between these two approaches depends on the specific needs of the application, whether the priority is high analytical accuracy or affordable, large-scale monitoring.

#### 4.1.3. Microfluidic Chips

By integrating sample pretreatment and detection functions on a single chip, microfluidic techniques support the automated on-site measurement of BAs, effectively improving the practical operability and detection credibility; chips made from PDMS can complete high-precision multi-target screening of complex food samples within one hour. Differing from conventional microfluidic forms, paper-based microfluidic analytical devices operate entirely through capillary action and need no external powerful supply, which allows them to analyze meat and seafood samples in approximately 10 min at a remarkably low cost, rendering them highly suitable for resource-limited conditions and daily self-testing scenarios.

**Figure 6:**
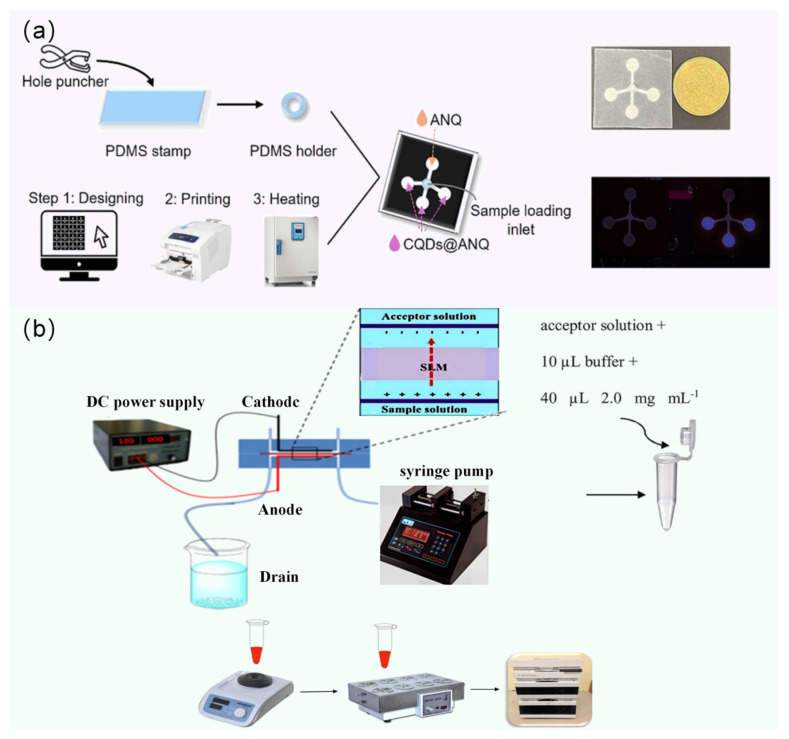
(**a**) Fabrication of a smartphone-integrated microfluidic paper-based platform for histamine determination in canned tuna. (Reprinted with permission from ref. [95]. Copyright 2024, Elsevier.) (**b**) An analytical procedure (electromembrane extraction + derivatization) developed based on a microfluidic chip system for the analysis of BAs in food. (Reprinted with permission from ref. [97]. Copyright 2018, Elsevier.).

Scholars have carried out several studies in recent years. He et al. [95] constructed a molecular imprinting-based microfluidic paper optical sensing system matched with smartphone equipment, which was applied to rapid on-site histamine measurement in canned tuna products (Figure 6a). Relevant experimental results showed that its linear detection interval covered 20–100 mg/kg, with the LOD reaching 14.04 mg/kg. Liu et al. [96] further exploited DNA hybridization and competitive binding mechanisms to fabricate a novel SERS-responsive DNA microfluidic chip, which can be reused for cyclic histamine testing. The system presented a detection limit of 1 × 10^−9^ g/mL in deionized water and 1 × 10^−8^ g/mL when detecting actual beer samples. Zarghampour et al. [97] reported an on-chip electromembrane extraction (CEME) method coupled with dabsyl chloride derivatization and high-performance liquid chromatography–ultraviolet detection for the simultaneous determination of five BAs (histamine, tryptamine, putrescine, cadaverine, and spermidine) in sausage and kielbasa samples (Figure 6b). This method exhibited detection limits of 3–8 μg/L.

The LODs for histamine are 14.04 mg/kg (He et al.), as low as 0.001 mg/L in water and 0.01 mg/L in beer (Liu et al.), and 3–8 μg/L (0.003–0.008 mg/L) for all five amines (Zarghampour et al.). For histamine, international regulatory maximum levels are 50–200 mg/kg. Even the highest LOD (14.04 mg/kg) is about 3.5-fold below the strictest limit (50 mg/kg), while the other methods are three to six orders of magnitude lower. For other BAs without statutory limits, the generally recommended total biogenic amine threshold is 100–200 mg/kg, which is far above the obtained sensitivities. Therefore, these microfluidic and extraction-based methods can reliably determine whether a food sample poses a safety risk or fails to meet regulatory requirements, and they provide sufficient sensitivity for on-site and laboratory surveillance.

In summary, these four studies demonstrate distinct optimization strategies for BA detection. Tang’s chip-mass spectrometry system delivers ultrahigh sensitivity and full automation but exhibits limited portability. Conversely, He’s smartphone–paper platform trades some sensitivity for a low-cost, power-free solution ideal for on-site screening. Liu’s SERS-DNA chip enables sensitive, reusable detection yet requires benchtop equipment like Tang’s approach. Zarghampour’s CEME-HPLC-UV system provides reliable laboratory-based performance with excellent sample clean-up and low solvent consumption, though its field applicability lacks the convenience of He’s paper-based device. Overall, a clear tradeoff exists between analytical performance and portable deployment ease. Future work should integrate high-sensitivity sensing technologies with compact, miniaturized devices to bridge this gap and broaden practical applications.

#### 4.1.4. Smartphone-Based Detection Devices

With the continuous iteration and upgrading of hardware and software facilities in recent years, smartphones have turned into an economical, portable, and easy-operated carrier for rapid on-site detection. These devices embed various sensing components to realize direct field data acquisition and real-time dynamic condition monitoring. Current smartphone-dependent detection technologies mostly adopt internal optical sensors such as colorimetric sensors, fluorescent sensors, chemiluminescent sensors, and dual-mode colorimetric–fluorescent sensors, and the abundant sensor types can well accommodate different actual monitoring demands [98].

Smartphone-based platforms leverage their internal high-resolution cameras as optical sensing units for quantitative signal acquisition, eliminating reliance on bulky laboratory equipment and enabling rapid on-site screening operations, typically completing an entire assay within 15–30 min from sample application. These integrated systems combine signal conditioning, data analysis, and visual presentation into a unified workflow, thus achieving real-time quantitative detection. Conventional paper-based sensors, in contrast, can only deliver semi-quantitative results by naked-eye observation, whereas smartphone-assisted detection provides substantially improved precision, yet the total turnaround time for both approaches remains comparable (usually 15–20 min for colorimetric strips, plus 1–3 min for image processing). Unlike pump-driven microfluidic chips and spectrometer-based SERS platforms, which often require 30–60 min per run due to complex fluidic control or spectral acquisition, smartphones paired with miniaturized potentiostats maintain stable performance while retaining excellent portability, and they can generate electrochemical readouts within 10–15 min of reaction completion.

### 4.2. Challenges in On-Site Detection and Commercialization Bottlenecks

The technical bottlenecks of on-site BA detection mainly derive from the inherent tradeoff between analytical performance and field applicability. Such challenges occur in multiple aspects. First, simplified pretreatment for portable rapid detection conflicts with the elimination of complex food matrix interference and non-specific binding, thereby restricting detection accuracy and specificity. Second, conventional biorecognition elements including enzymes and antibodies suffer from poor environmental stability under field conditions, which hinders the development of low-cost, high-affinity, and broad-spectrum recognition receptors. Moreover, signal-to-noise ratio and cost limitations make it difficult to simultaneously realize low detection limits, wide linear ranges, and multi-target detection that conform to regulatory standards. Such obstacles hinder standardized field operations and dependable quality control procedures. Moreover, they compromise the reproducibility and precision of test outcomes, ultimately complicating the commercialization of relevant detection methodologies. Table 7 presents the adaptability of recognition elements and platforms for BA detection in different food matrices.

## 5. Summary and Future Perspectives

### 5.1. Summary

As public awareness around food quality and safety continues to grow, detection methods for BAs are gradually shifting from conventional laboratory-based assays toward analytical approaches that are not only faster but also field-friendly. This review offers a comprehensive survey of detection technologies built upon different recognition elements. We explore established biological receptors, including antibodies and enzymes, alongside more recent biomimetic and synthetic materials like aptamers, MIPs, peptides, and nanoenzymes. For each recognition medium, we examine the underlying working mechanisms, assess their analytical performance, and discuss the various forms in which they can be deployed. The paper also delves into how molecular recognition events are converted into measurable signals, covering colorimetric, fluorescent, chemiluminescent, and electrochemical outputs. Finally, we examine whether these sensing units can be effectively integrated into portable devices such as lateral flow strips, microfluidic chips, and smartphone-based detectors.

### 5.2. Future Trends

This field has four viable avenues for future research. First, to create robust recognition elements adaptable to harsh environments, we must combine experimental biotechnology with computational design. Techniques including protein engineering, directed evolution, and computer-aided optimization (e.g., machine learning-guided mutagenesis) can significantly improve the resistance of enzymes and nanobodies to heat, extreme pH, and protease degradation. Optimized SELEX protocols and composite biomimetic materials also enable aptamers to achieve both high-affinity target recognition and built-in signal amplification. Looking ahead, in silico prediction of binding stability under stress conditions—using deep learning models trained on structural databases—will accelerate the discovery of next-generation bioreceptors, reducing the need for costly trial-and-error screening and ensuring that the recognition layer itself is intrinsically more tolerant to real-world interferences.

Second, cross-responsive sensor arrays offer a powerful platform for simultaneous, accurate detection of multiple BAs, but their full potential hinges on advanced data interpretation. Incorporating machine learning is no longer optional; it is the key to decoding the complex, overlapping “fingerprint patterns” generated by these arrays. To achieve reliable accuracy in real-world, non-standard environments (with fluctuating temperature, humidity, and interfering gases), we propose the deployment of 1-D Convolutional Neural Networks (1D-CNNs) and attention-based transformers that learn the temporal evolution of sensor responses. Unlike conventional chemometric methods, these models can disentangle target signals from environmental noise by exploiting transient dynamics. Crucially, domain adaptation techniques must be embedded to bridge the gap between laboratory-calibrated data and field measurements, allowing the model to generalize without full recalibration. Moreover, Bayesian neural networks should be adopted to output not only a predicted concentration but also a confidence interval: when uncertainty exceeds a threshold, the system automatically flags the data as unreliable and triggers a self-check routine. This uncertainty-aware framework transforms the array into a resilient analytical tool that can maintain diagnostic performance even when fingerprint patterns are distorted by unpredictable environmental fluctuations.

Third, wearable detectors and flexible IoT sensors work as a collaborative system to provide continuous real-time monitoring of temperature, humidity, gas release, and other critical parameters across the entire food processing, transportation, and storage chain. These devices—including printable sensing patches, smart gloves, and conductive-ink-based electrochemical sensors—can be embedded in packaging materials, production machinery, and transport vessels for non-invasive monitoring without disrupting routine operations. Data are wirelessly transmitted to cloud platforms, granting remote access to real-time information and enabling automatic early-warning protocols when preset thresholds are exceeded [99]. Low-power wearable tools, such as biosensor-integrated gloves and peroxide-sensitive printed electrodes, allow for in situ detection of hazardous pathogens and chemical residues during food manufacturing [100] and can connect with mobile phones or data terminals for on-site wireless analysis, offering instant guidance to operators. To overcome bandwidth limitations and signal drift in such distributed networks, future IoT nodes should incorporate edge-computing capabilities with lightweight AI models for on-device pre-processing, while federated learning allows for collaborative model improvement across nodes without sharing raw data. A closed-loop recalibration mechanism, triggered by uncertainty estimates from on-board Bayesian inference, further ensures reliable performance throughout the logistic chain. Finally, a scenario-oriented comparison of different food matrices will provide practical criteria for selecting appropriate recognition elements and portable platforms, bridging the gap between sensor design and end-user needs.

Finally, to tackle engineering barriers and boost commercial deployment, simplified plug-and-play microfluidics, standardized protocols, and scalable fabrication methods are essential. Harmonizing data formats and communication interfaces ensures interoperability across different sensor platforms. Modular, cartridge-based designs with pre-loaded recognition elements reduce user intervention, while cost-effective manufacturing enables customization. These hardware advances, coupled with user-friendly software embedding the aforementioned AI models, will facilitate the transition from laboratory prototypes to field-ready systems for food safety.

## Figures and Tables

**Table 1:** Common biogenic amines, regulatory limits, main food matrices, and typical detection technologies.

Biogenic Amine	Major Regulatory Limits (mg/kg)	Main Food Matrices	Typical Detection Technologies
Histamine	China: high-histamine fish ≤ 400; other marine fish ≤ 200 USA (FDA): aquatic products ≤ 50 EU: Scombridae/tuna ≤ 100	High-histamine marine fish (tuna, mackerel, and saury), fish sauce, fermented fish products, wine, and aged cheese	Industrial: HPLC with pre-column derivatization (DNS-Cl/benzoyl chloride)—GB national standard, LC-MS/MS (direct injection, no derivatization) as confirmatory method, and commercial ELISA. Proof-of-concept: electrochemical biosensors, fluorescent probes, and paper-based sensors (still in lab development).
Putrescine	_	Aquatic products (fish and shrimp), meat, fermented soy products, and beer	Industrial: HPLC, LC-MS/MS, and ion chromatography (direct injection, suitable for high-throughput screening). Proof-of-concept: self-assembled dipeptide-based ratiometric fluorescent nanoprobes (lab research).
Cadaverine	_	Meat, aquatic products, and fermented vegetables	Industrial: HPLC-DAD and LC-MS/MS (good reproducibility). Proof-of-concept: portable colorimetric test strips (BCY-OH probe) and paper-based fluorescent labels.
Tyramine	USA: other foods < 100 EU: 100–800 for certain fermented products	Cheese, fermented sausages, wine, and fish	Industrial: HPLC-UV/DAD, LC-MS/MS (often co-analyzed with histamine), and ion-exchange chromatography. Proof-of-concept: fluorescence immunoassay using multicolor UCNPs—validated in lab.
Tryptamine	_	Dairy products, meat, and fermented foods	Proof-of-concept: Only a few HPLC studies; very few novel sensing reports—still fundamental research.
Spermidine	_	Meat, dairy, soy products, ad cereal germs	Proof-of-concept: Polyamine oxidase sensors, most sensor technologies are at the laboratory stage.
Spermine	_	Animal offal, meat, legumes, and cereals	Proof-of-concept: Similar to spermidine, lacking field validation.
β-Phenylethylamine	_	Chocolate, wine, cheese, and fermented meat products	Proof-of-concept: Only chromatographic methods; sensing technology is largely absent.
Octopamine	_	Fish and citrus products	Proof-of-concept: Very few studies; existing sensors suffer from poor selectivity.

**Table 2:** Antibody recognition system-based sensing strategies for BA detection in foods.

BAs	Sensor Architecture	Transducer	Sample	LOD	Analytical Range	Reference
Tyramine and histamine	Competitive magnetic separation immunological method: bicolor upconversion probe and magnetic-coated antigen	Multiple fluorescence immunoassay	9 food sample types	0.1 μg/L, 0.01 μg/L	0.5–100 μg/L, 0.1–100 μg/L	[15]
Tyramine	Microplate coated with coating antigen, with competitive immunoreaction and ALP-induced AuNS growth for colorimetric signal via smartphone	Colorimetric signal	Beef, pork, and yogurt	19.7 mg/kg	0.313–20 mg/L	[16]
Histamine	Ratiometric fluorescence immunoassay driven by nanozyme, with histamine-specific antibodies tagged by Prussian blue nanoparticles	Fluorescence based on carbon dots	Cod fish, red wine, and yogurt	1.2 ng/mL	1.6 ng/mL–125 µg/mL	[17]
Tyramine	Microplate coated with Tyr–BSA, incorporating HRP-labeled anti-Tyr mAb and using TMB colorimetric detection	Colorimetric signal	Fish samples	2 ng/mL	5–40 ng/mL	[18]
Histamine	Flexible screen-printed electrode coated with oxygen plasma-treated SWCNTs, direct competitive immunoassay	Chronoamperometric	Fish samples	2.48 pg/mL	0.005–50 ng/mL	[19]
Histamine	Competitive lateral flow immunoassay (LFIA) using superparamagnetic iron oxide nanoparticles as labels	Magnetic and colorimetric	Red wine	1.2 mg/L, 1.5 mg/L	1–100 mg/L	[20]

**Table 3:** Aptamer recognition system-based sensing strategies for BA detection in foods.

BAs	Sensor Architecture	Transducer	Sample	LOD	Analytical Range	Reference
Histamine	Fluorescent structure-switching aptasensor based on a Cy5-labeled aptamer paired with a quencher DNA strand	Fluorescence of Cyanine 5	Tuna samples	1 µM	1–1000 µM	[28]
Spermine	Spermine aptamer (APJ-6) selected by Capture-SELEX, used as recognition probe in fluorescence-based assay	Fluorescence	Pork samples	0.052 nM	0.1–20 nM	[29]
Tryptamine	Magnetic core-satellite SERS aptasensor uses Fe_3_O_4_@Au-apt and AuNP-4MBN-cDNA probes; target binding releases signal probes to reduce SERS	SERS in the “biological-silent” region	Liquor, white wine, and vinegar	0.39 × 10^−3^ mg/L	0.001–100 mg/L	[30]
Histamine Tryptamine	Competitive colorimetric aptamer detection: biotinylated aptamers competitively bind to the target, with HRP catalyzing the TMB-H_2_O_2_ colorimetric reaction	Competitive colorimetric detection	Pork and fish	1 ng/mL, 10 ng/mL	1–100 ng/mL, 10–1000 ng/mL	[26]
Histamine Tyramine	A dual-donor fluorescence resonance energy transfer aptamer sensor, featuring a quantum dot donor and a MoS_2_ receptor	Fluorescence (fluorescence quenching and recovery)	Salmon and mackerel	0.1 ng/mL, 0.25 ng/mL	0.5–100 ng/mL	[27]
Histamine	PF8BT polymer dots conjugated with histamine-specific aptamer, Cy3 as acceptor, and FRET-based ratiometric probe	Fluorescence	Aquaculture water and tuna	0.38 μmol/L	3–21 μmol/L	[31]
Dopamine	Electrochemical aptasensor using Ce-MOF modified electrode, S1-AP double strand, and m-PdNFs-S2-G4-MBs signal probe	Square wave voltammetry	Human serum (not yet validated in food matrices)	6 pM	6 pM–100 nM	[32]
β-Phenethylamine	DNA-assembled Au dumbbell dimers with aptamer and 4-MBN reporter; target binding narrows nanogap to activate SERS hotspots	Surface-enhanced Raman	Stinky tofu, coffee, and soy sauce	3.8 × 10^−7^ mg/L	10^−6^–10^−2^ mg/L	[33]

**Table 4:** MIP recognition system-based sensing strategies for BA detection in foods.

BAs	Sensor Architecture	Transducer	Sample	LOD	Analytical Range	Reference
Tyramine	MPt/MIP as adsorbent for dispersive solid-phase extraction; magnetic screen-printed carbon electrode (MSPCE) for collection via external magnetic field	Differential pulse voltammetry	Yogurt	3.8 ng/mL	8.0–380.0 ng/mL	[43]
Histamine and tyramine	MIPs synthesized via surface polymerization on SiO_2_ carriers; CdSe/ZnS quantum dots embedded as fluorescent probes	Fluorescence spectroscopy	Cheese	14.57 μmol/L	0.01–10 mmol/L	[44]
Histamine and tryptamine	SiO_2_@AuNPs core–shell with polydopamine imprinted layer; functions as both enrichment adsorbent and SALDI-MS matrix	Surface-assisted laser desorption/ionization time-of-flight mass spectrometry	Beer, sausage, and chicken	0.2 ng/mL, 0.1 ng/mL	1–500 ng/mL, 0.8–300 ng/mL	[45]
Histamine	Bulk-polymerized MAA particles, OC_1_C_10_-PVV adhesive	Colorimetric	PBS buffer solution (not validated in food matrices)	15 nM	500–1000 nM	[46]
Tryptamine	Azide-functionalized COFs prepared via Schiff base and click chemistry, combined with MIPs for tryptamine recognition	Fluorescence	Meat products	1.74 μg/L	3–120 μg/L	[47]
Histamine	MIP film electropolymerized on PEDOT-modified gold inverse opal electrode; semi-covalent imprinting approach	Electrochemical (CV and EIS)	Seafood	1.07 nM	50 nM–500 μM	[48]
Octopamine	Core–shell upconversion nanoparticle @ZIF-8@ molecularly imprinted polymer fluorescence sensor	Fluorescence (upconversion luminescence)	Beer	0.081 mg/L	0.1–10 mg/L	[40]
Histamine	AuNPs electrodeposited on GCE, followed by L-cysteine self-assembly and electropolymerized MIP layer	Differential pulse voltammetry	Beer and wine	0.6 μM	1.0–40.0 μM, 40.0–107.0 μM	[41]
Tyramine	MISPE sample pretreatment coupled with electrochemical sensor: SPCE modified with PEDOT	Voltammetric	Milk	0.00231 µM	5–100 nM	[49]
Histamine	In situ electropolymerized MIP film on cysteamine-modified gold screen-printed electrode	Electrochemical (EIS and CV)	Sardine and mackerel	210 nM	500 nM–1 mM	[50]
Tryptamine	MIP based on carbon dot-embedded covalent organic frameworks; fluorescence quenching upon target binding	Fluorometric with luminescent carbon dots	Meat samples	7 µg/kg	0.025–0.4 mg/kg	[51]
Tyramine	Pt disk electrode coated with MIP film via oxidative electropolymerization	Electrochemical (DPV and EIS) with Fe(CN)_6_^4−^/^3−^ redox probe	Mozzarella cheese whey	159 μM, 168 μM	290 μM–2.64 mM	[52]
Putrescine	MIP chromogenic hydrogel (PVA/ninhydrin); imprinting with imitate templates and color development upon putrescine vapor exposure	Colorimetric	Putrescine aqueous solution (not validated in food matrices)	4 mg/g	4–24 mg/g	[53]

**Table 5:** Enzyme recognition system-based sensing strategies for BA detection in foods.

BAs	Sensor Architecture	Transducer	Sample	LOD	Analytical Range	Reference
Putrescine	Glassy carbon electrode modified with clay, incorporating physically adsorbed unfolded hemoglobin and glutaraldehyde-crosslinked DAO	Electrochemical	Pork samples	3.3 × 10^−12^ mol/L	1.0 × 10^−11^–1.0 × 10^−10^ mol/L 1.0 × 10^−10^–1.0 × 10^−9^ mol/L	[67]
Tyramine	Unmodified electrode; direct electrochemical detection of dopamine generated in situ from tyramine oxidation by tyrosinase	Electrochemical (cyclic voltammetry and square wave voltammetry)	Yogurt and human urine	0.69 μM	5.0–100 μM, 1.0–100 μM	[68]
Histamine	Screen-printed carbon electrode with enzyme immobilized via glutaraldehyde/BSA crosslinking	Amperometric (chronoamperometry)	Fish extracts	0.5 mg/L	1–75 mg/L	[69]
Tyramine	Tyrosinase/AuNPs/Nb_2_CT_x_ MXene functionalized W-shaped LSPR optical fiber	W-shaped LSPR optical fiber	Agricultural and food samples	6.96 µM	0–100 µM	[70]
Cadaverine, putrescine, tyramine, and histamine	Carbon paste electrode (CPE) modified with MnO_2_, incorporating PSAO immobilized by a Nafion^®^ membrane	Electrochemical	Commercial fish sauce	10 μg/mL, 8 μg/mL	30–88 μg/mL, 24–67 μg/mL	[71]

**Table 6:** Peptide recognition system-based sensing strategies for BA detection in foods.

BAs	Sensor Architecture	Transducer	Sample	LOD	Analytical Range	Reference
Histamine, tyramine, putrescine, tryptamine, and spermine	Ratiometric fluorescent nanoprobe based on self-assembled dipeptide combined with methyl red, producing dual-emission peaks at 400 nm and 610 nm.	Ratiometric fluorescence (dual-emission ratio change)	Meat products	0.38 mg/L, 0.37 mg/L, 0.30 mg/L, 0.40 mg/L, 0.51 mg/L	1–50 mg/L	[73]
Tryptamine	FS@COPs: diphenylalanine (FF)-Zn^2+^ self-assembled fluorescent nanomaterial (FS) confined within covalent organic polymers (COPs) via in situ growth or encapsulation.	Fluorescence (excitation 360 nm, emission 450 nm), signal-off mode upon tryptamine binding	Real food samples	63.08 μg/kg	40–900 μg/L	[74]
Tyramine	Cuvette-type LSPR sensing chip: glass substrate coated with AuNPs, conjugated with TYR1 peptide via physical adsorption.	Localized surface plasmon resonance (LSPR); absorbance measured by UV-Vis spectrophotometer.	Beef, mackerel, and cheddar cheese	0.63 ppb, 0.46 ppb, 0.27 ppb	0.01–10 ppb, 0.01–10 ppm	[75]
Histamine	Glassy carbon electrode (GCE) modified with electrodeposited 1-amino-2-naphthol-4-sulfonic acid (ANSA), covalently immobilized with HBP via sulfonamide bond.	Electrochemical	Not tested in real samples (method development only; stated ready for wine/fermented beverages)	6.53 nM	10 nM–1 mM	[76]

**Table 7:** Adaptability of recognition elements and platforms for BA detection in different food matrices.

Food Category	Major BAs	Recommended Recognition Elements	Recommended Detection Platforms	Main Challenges
Aquatic products	Histamine, cadaverine, and putrescine	Antibodies, aptamers, and MIPs	Lateral flow strips, fluorescence sensors, and smart labels	Complex matrix (high protein/fat), rapid spoilage dynamics, and high risk of histamine poisoning
Fermented foods	Tyramine and histamine	MIPs, enzymes, and aptamers	Electrochemical electrodes and colorimetric arrays	High salt, pH fluctuations, interference from microbial metabolites, and pigment effects
Meat products	Putrescine, cadaverine, and tyramine	Enzymes, MIPs, and array-based sensors	Smart labels and colorimetric sensor arrays	Coexistence of multiple amines, insufficient specificity, and simultaneous accumulation of various amines during spoilage
Alcoholic beverages	Histamine and tyramine	MIPs and aptamers	Fluorescent probes and electrochemical chips	Interference from ethanol, organic acids, and pigments; pretreatment often required
Egg products	Putrescine and cadaverine	MIPs and enzymes	Paper-based colorimetric/fluorescence assays	Protein interference and high sample viscosity
Dairy products	Tyramine, histamine, and cadaverine	Antibodies, enzymes, and aptamers	Electrochemical immunosensors and microfluidic chips	Interference from fat globules and casein; complex lactic acid bacterial metabolism
Soy-based fermented products	Tyramine and putrescine	MIPs and array-based sensors	Smart labels and colorimetric arrays	High salt content, diverse microbial communities, and abundant free amino acids
Condiments	Histamine and tyramine	Aptamers and MIPs	Fluorescence/electrochemical paper-based sensors	Dark color, high salt concentration, and non-specific adsorption

## Data Availability

No new data were created or analyzed in this study. Data sharing is not applicable to this article.
